# Predictive performance of the PSU-neonatal early warning score in identifying newborns requiring intensive care

**DOI:** 10.3389/fped.2026.1804592

**Published:** 2026-05-28

**Authors:** Praenapa Chaithaweesup, Gunlawadee Maneenil, Anucha Thatrimontrichai, Manapat Praditaukrit, Pattima Pakhathirathien, Praew Chareesri, Supaporn Dissaneevate, Anong Chumgoon

**Affiliations:** 1 Division of Neonatology, Department of Pediatrics, Faculty of Medicine, Prince of Songkla University, Songkhla, Thailand; 2 Neonatal Moderate Care Unit, Nursing Services Division, Songklanagarind Hospital, Songkhla, Thailand

**Keywords:** early warning score, early warning system, intensive care, newborn, risk stratification

## Abstract

**Introduction:**

The Prince of Songkla University (PSU)-neonatal early warning score (PSU-NEWS) was developed to assist nurses in rapidly assessing the neonatal status and communicating clinical concerns. This study aimed to evaluate the accuracy of the PSU-NEWS in identifying neonates who required non-invasive ventilation (NIV) or admission to the neonatal intensive care unit (NICU).

**Methods:**

This retrospective study included neonates admitted to a nursery between July 2023 and June 2024. All infants were born at ≥35 weeks’ gestation and birth weight ≥1,800 g and were categorized into those transferred to the NICU and those transferred to the postpartum ward. The first and second PSU-NEWS assessments were performed 1–2 h and 4–6 h after birth.

**Results:**

The first and second PSU-NEWS were assessed in 2,191 and 1,908 neonates, respectively. Of these, 176 required NICU admission and 132 required NIV. In predicting NICU admission, the first PSU-NEWS demonstrated an overall AUC of 0.637 (95% CI: 0.593–0.681). At a cut-off score of ≥2, the receiver operating characteristic (ROC) analysis yielded an AUC of 0.628 with a sensitivity of 47% and specificity of 78%, whereas the second PSU-NEWS showed an overall AUC of 0.755 (95% CI: 0.717–0.792). At a cut-off score of ≥1, the ROC analysis yielded an AUC of 0.751 with a sensitivity of 60% and specificity of 90%. For predicting NIV requirement, the first PSU-NEWS demonstrated an overall AUC of 0.628 (95% CI: 0.575–0.682). At a cut-off score of ≥2, the ROC analysis yielded an AUC of 0.632 with a sensitivity of 48% and specificity of 78%, whereas the second PSU-NEWS showed an overall AUC of 0.785 (95% CI: 0.744–0.826). At a cut-off score of ≥1, the ROC analysis yielded an AUC of 0.783 with a sensitivity of 67% and specificity of 89%.

**Conclusions:**

The PSU-NEWS demonstrated moderate accuracy in identifying neonates requiring NICU admission or NIV, and the second assessment provided a better predictive performance than the first. Serial scoring may enhance early recognition of infants requiring intensive care.

## 1 Introduction

Early warning scores (EWSs) are increasingly integrated into pediatric care as structured tools to support the early recognition of clinical deterioration (1, 2). By providing objective physiology-based assessments, EWSs enhance situational awareness, facilitate timely communication among healthcare professionals, and promote patient safety across various levels of clinical experience and workload (3–5).

The neonatal period is a uniquely vulnerable phase characterized by a rapid physiological transition from fetal to postnatal life (6). During this period, clinical deterioration may present with subtle or nonspecific signs and can progress rapidly, making early identification challenging. Delayed recognition of illness in neonates is associated with increased morbidity and demands high-level care, including respiratory support and admission to the neonatal intensive care unit (NICU). Therefore, reliable early warning systems tailored to neonatal physiology are essential for optimizing early intervention and appropriate triage.

To date, evidence supporting the use of EWSs in neonatal populations remains limited, and no universally accepted neonatal EWS has been established (7). Several neonatal early warning systems have been developed within specific institutional contexts and evaluated for their ability to predict NICU admission or the need for clinical interventions, such as intravenous therapy, respiratory support, and antibiotic administration. However, variations in the included parameters, target populations, and outcome measures limit the generalizability and comparability of these tools (3).

Previously reported systems include the neonatal trigger score (NTS), which incorporates heart rate, temperature, respiratory rate, respiratory distress, level of consciousness, and prefeed blood glucose (7). The newborn early warning system for term neonates weighing >2.5 kg (8, 9) and the newborn early warning trigger and track (NEWTT) system were introduced by the British Association of Perinatal Medicine for high-risk neonates (3). In addition, the Cardiac Children's Hospital Early Warning Score (C-CHEWS), developed at Boston Children's Hospital, integrates physiological and clinical indicators relevant to pediatric cardiac patients (10, 11). While these tools demonstrate the feasibility of neonatal EWS, their applicability to broader neonatal populations and their ability to predict clinically meaningful outcomes remain uncertain.

Songklanagarind Hospital, a tertiary university hospital of Prince of Songkla University in southern Thailand, manages more than 2,500 live births annually. Prior to 2023, there was no standardized neonatal early warning system implemented in our nursery, with clinical monitoring relying primarily on routine vital signs and individual clinical judgment. In response to the need for a context-specific and objective monitoring tool, the PSU-Neonatal Early Warning Score (PSU-NEWS) was developed and implemented in the nursery setting. The PSU-NEWS is designed for neonates born at least 35 weeks of gestation with a birth weight of at least 1,800 g and comprises five physiological parameters: heart rate (HR), respiratory rate (RR), body temperature (BT), blood pressure (BP), and oxygen saturation (SpO_2_), together with an assessment of the level of consciousness, predefined escalation criteria, and standardized team responses. This study aimed to evaluate the accuracy of PSU-NEWS in identifying neonates requiring escalation of care, specifically NICU admission and the need for non-invasive ventilation (NIV).

## 2 Materials and methods

### 2.1 Study design and population

This retrospective cohort study included neonates admitted to a nursery at Songklanagarind Hospital, a major tertiary referral center in Southern Thailand, between July 2023 and June 2024. A consecutive sampling method was employed to recruit all eligible participants who met the selection criteria during the study period. The inclusion criteria were: (1) infants born at a gestational age of ≥35 weeks, and (2) a birth weight of ≥1,800 g. Neonates were excluded from the study if they: (1) were transferred to the NICU before the PSU-NEWS assessment, or (2) had incomplete data to calculate the PSU-NEWS score.

Ethical approval was obtained from the Institutional Review Board and Ethics Committee of the Faculty of Medicine, Prince of Songkla University, Thailand (REC 67-473-1-1). The requirement for informed consent was waived.

### 2.2 Nursery and NICU management protocol

**Figure 1:**
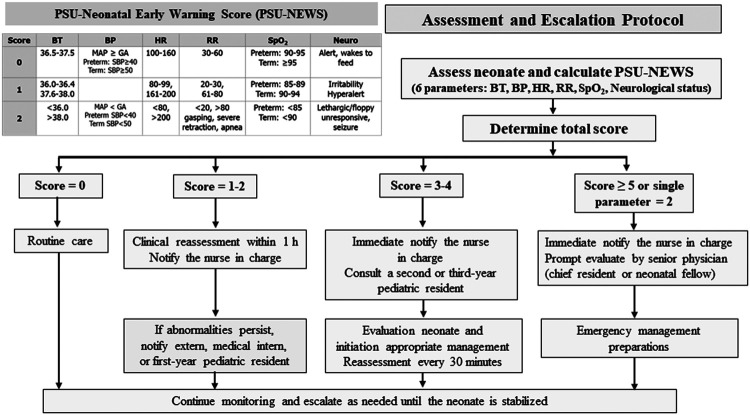
PSU-Neonatal early warning score BT, body temperature; BP, blood pressure; MAP, mean arterial pressure; GA, gestational Age; SBP, systolic blood pressure; HR, heart rate; RR, respiratory rate; SpO_2_, oxygen saturation; neuro, neurological status.

The PSU-NEWS was developed by a multidisciplinary neonatal care team (Figure 1). The score comprises six clinical parameters, each assigned a score ranging from 0 to 2. Individual parameter scores were summed to generate a cumulative score ranging from 0 to 12, with higher scores indicating a greater deviation from normal physiological status. PSU-NEWS assessments were performed by trained nurse at 1–2 hours and 4–6 hours of life in the nursery.

The clinical management and escalation followed a predefined protocol. Neonates with a PSU-NEWS score of 0 received routine care according to standard nursery practices. For total scores of 1–2, clinical reassessment was conducted within 1 hour, and the nurse in charge was notified. If the abnormalities persisted, escalation to a junior physician, including the extern, medical intern, or first-year pediatric resident, was initiated. A total score of 3–4 required immediate notification by the nurse in charge and consultation with a second- or third-year pediatric resident to evaluate the neonate and initiate appropriate management. Clinical reassessments were performed every 30 minutes until stabilization or further escalation of care was achieved. For neonates with a total score of ≥5 or with any single parameter scored 2, immediate escalation was mandated, including prompt evaluation by a senior physician (chief resident, or neonatal fellow). Emergency management preparations, including resuscitation readiness and emergency equipment, were undertaken simultaneously.

The final disposition decisions were based on the attending physician's clinical assessment, independent of the PSU-NEWS. Clinically stable neonates were transferred to the postpartum ward, whereas neonates identified as unwell were transferred to the NICU for further management.

The institutional criteria for NICU admission included neonates from birth to 30 days of age, as well as infants older than 30 days with a body weight <2,000 g who required continuous close monitoring and intensive care. Additional admission criteria included preterm infants with a gestational age <34 weeks and a birth weight <1,800 g. For neonates with a gestational age >35 weeks (the focus of this study), admission was primarily indicated by unstable vital signs, respiratory distress requiring respiratory support, or the need for continuous intensive monitoring, congenital heart disease requiring close monitoring or surgical intervention, and those requiring surgical management, total or partial blood exchange transfusion, or treatment in an isolation room.

After admission to the NICU, neonates with clinical signs of respiratory distress (e.g., tachypnea, retractions, or grunting) were initiated on NIV. The specific type of NIV such as noninvasive positive pressure ventilation (NIPPV), continuous positive airway pressure (CPAP), bilevel positive airway pressure (BiPAP), nasal high-frequency oscillatory ventilation (nHFOV), or high-flow nasal cannula (HFNC), was selected at the discretion of the attending physician based on the patient's clinical condition.

### 2.3 Data collection

Neonatal data were extracted from the hospital's electronic medical record system and included gestational age, birth weight, sex, Apgar scores, use of NIV, length of hospital stay, and discharge diagnoses. All PSU-NEWS records, including the timing of the assessments and any interventions initiated at the time of scoring, were systematically reviewed and recorded.

### 2.4 Statistical analysis

Statistical analyses were performed using the Epicalc package in R software (version 4.4.1; R Foundation for Statistical Computing, Vienna, Austria). Categorical variables were summarized as percentages and compared using the chi-square test. Continuous data were presented as mean ± standard deviation (SD) or median (interquartile range, IQR) and were analyzed using the Wilcoxon rank-sum test. Receiver operating characteristic (ROC) curve analysis was conducted to determine optimal PSU-NEWS cutoff values for predicting NICU admission and the need for NIV.

## 3 Results

A total of 2,484 neonates were born at Songklanagarind Hospital during the study period, of which 2,191 met the inclusion criteria and were enrolled, while 293 were excluded because they were transferred before completion of the PSU-NEWS assessments. The first and second PSU-NEWS assessments were completed in 2,191 and 1,908 neonates, respectively. The second assessment was not available in 283 neonates, primarily due to early transfer to the postpartum ward or NICU before the scheduled second assessment.

The study population had a median (IQR) gestational age of 38 (38–39) weeks, ranging from 35 to 41 weeks. Among these, 158 neonates (7.2%) were late-preterm (35 to <37 weeks of gestation). The proportion of late-preterm neonates was significantly higher in the NICU group compared to the postpartum ward group (18.2% vs. 6.3%, *p* < 0.001). The median (IQR) birth weight was 3,058 (2,791–3,315) g, with a minimum of 1,804 g and a maximum of 4,480 g; a total of 182 neonates (8.3%) were classified as having low birth weight (<2,500 g). The proportion of low birth weight neonates in the NICU group was higher than in the postpartum ward group (13.1% vs. 7.9%, *p* = 0.025). Approximately half of the participants were male (50.5%), and 61.5% were delivered via cesarean section. Overall, 176 neonates (8.0%) required NICU admission. Compared to those transferred to the postpartum ward, neonates admitted to the NICU had a significantly higher proportion of males (64.2% vs. 49.3%, *p* < 0.001) and a significantly longer median length of hospital stay (6 vs. 4 days, *p* < 0.001). Additionally, gestational age and Apgar scores at 1 and 5 minutes were significantly lower in the NICU group (all *p* < 0.001). Detailed demographic and clinical characteristics are presented in Table 1.

**Table 1:** Demographic and clinical characteristics of neonates.

Baseline characteristics	Total (*n* = 2191)	Neonates transferred to NICU (*n* = 176)	Neonates transferred to postpartum ward (*n* = 2015)	*P-*value
GA, weeks^a^	38 (38–39)	38 (37–39)	38 (38–39)	0.001
Min, Max	35, 41			
GA <37 weeks, n (%)	158 (7.2)	32 (18.2)	126 (6.3)	<0.001
Male sex, n (%)	1107 (50.5)	113 (64.2)	994 (49.3)	<0.001
Birth weight, g^a^	3058 (2791–3315)	3010 (2715–3291)	3059 (2799–3316)	0.23
Min, Max	1804, 4480			
Birth weight <2500 g, n (%)	182 (8.3)	23 (13.1)	159 (7.9)	0.025
Apgar score at 1 min^a^	9 (8–9)	8 (8–9)	9 (8–9)	<0.001
Apgar score at 5 min^a^	9 (9–9)	9 (9–9)	9 (9–9)	<0.001
Cesarean section, n (%)	1348 (61.5)	112 (63.6)	1236 (61.3)	0.60
Indication cesarean section, n (%)				0.99
Previous cesarean section	608 (45.1)	55 (49.1)	553 (44.7)	
Cephalopelvic Disproportion	203 (15.1)	18 (16.1)	185 (15)	
Failed induction	188 (13.9)	11 (9.8)	177 (14.3)	
Fetal distress	83 (6.2)	6 (5.4)	77 (6.2)	
Meconium stain amniotic fluid	38 (2.8)	4 (3.6)	34 (2.8)	
Twins pregnancy	46 (3.4)	5 (4.5)	41 (3.3)	
Placenta previa	31 (2.3)	4 (3.6)	27 (2.2)	
Other	151 (11.2)	9 (8)	142 (11.5)	
Primary diagnosis, n (%)				
TTNB		115 (65.3)		
MAS		17 (9.7)		
Neonatal sepsis		3 (1.7)		
Hypoglycemia		9 (5.1)		
Other		32 (18.2)		
Requiring NIV, n (%)		132 (75)		
Requiring IMV, n (%)		10 (5.7)		
LOS, day^a^	4 (4–5)	6 (4–8)	4 (4–5)	<0.001

Categorical variables are presented as n (%) and analyzed using the Chi-square test or Fisher's exact test. GA, gestational age; IMV, invasive mechanical ventilation; LOS, length of hospital stays; MAS, meconium aspiration syndrome; NIV, non-invasive ventilation; TTNB, transient tachypnea of the newborn.

a Data are presented as median (interquartile range) and analyzed using the Wilcoxon rank-sum test.

Regarding the mode of delivery, 61.5% of neonates were delivered via cesarean section. The most common indication was a previous cesarean section (45.1%), followed by cephalopelvic disproportion (15.1%) and failed induction (13.9%). Fetal-related indications, including fetal distress and meconium-stained amniotic fluid, were present in 6.2% and 2.8% of the cesarean section cases, respectively (Table 1).

Among neonates admitted to the NICU, the most common discharge diagnosis was transient tachypnea of the newborn (65%), followed by meconium aspiration syndrome (10%), hypoglycemia (5%), neonatal sepsis (2%), and other diagnoses (18%). A total of 132 neonates (75%) required NIV for a median (IQR) duration of 3 (2–4) days. In addition, 10 neonates (5.7%) required invasive mechanical ventilation, with a mean ± SD duration of 3.6 ± 1.2 days.

 Table 2 summarizes the first and second PSU-NEWS scores among neonates transferred to the NICU and those remaining in the postpartum ward. The median (IQR) PSU-NEWS scores at both assessment time points were significantly higher in the NICU transfer group. In this group, a PSU-NEWS score ≥5 or the presence of a single parameter scored 2 was observed in 47/176 (26.7%) neonates at the first assessment and 17/176 (9.7%) at the second assessment. In comparison, elevated scores in the postpartum ward group were observed in 367/2015 (18.1%) neonates at the first assessment, decreasing to 21/1732 (1.2%) neonates at the second assessment. Regarding the specific parameters, abnormal findings in the NICU group during the first assessment were most frequently associated with BT (83%) and SpO_2_ (10.6%). Similarly, in the postpartum ward group, the vast majority of elevated first assessment scores were due to abnormal BT (99%), with only 1% related to SpO_2_. In the second assessment, elevated scores in the NICU group were primarily attributable to abnormalities in BT (53%) and RR (35%). In the postpartum group, elevated scores at the second assessment remained predominantly related to BT (95%), followed by RR (5%).

**Table 2:** First and second PSU-NEWS.

PSU-NEWS	Neonates transferred to NICU	Neonates transferred to postpartum ward	*P*-value
**First PSU-NEWS, n**	176	2015	
Median total score	1 (1–2)	1 (0–1)	<0.001
Total score, n/N (%)			<0.001
0	31/176 (17.6)	542/2015 (26.9)	
1	62/176 (35.2)	1038/2015 (51.5)	
2	67/176 (38.1)	408/2015 (20.2)	
3	14/176 (8)	27/2015 (1.3)	
4	2/176 (1.1)	0 (0)	
Total score ≥5, with one item scoring 2, n/N (%)	47/176 (26.7)	367/2015 (18.2)	0.008
**Second PSU-NEWS, n**	176	1732	
Median total score	1 (0–1)	0 (0–0)	<0.001
Total score, n/N (%)			<0.001
0	70/176 (39.8)	1557/1732 (89.9)	
1	78/176 (44.3)	149/1732 (8.6)	
2	18/176 (10.2)	25/1732 (1.4)	
3	9/176 (5.1)	1/1732 (0.1)	
4	1/176 (0.6)	0 (0)	
Total score ≥5, with one item scoring 2, n/N (%)	17/176 (9.7)	21/1732 (1.2)	<0.001

The distribution of individual PSU-NEWS parameters further clarifies the nature of the elevated scores (Table 3). At the first assessment, BT showed the highest frequency of abnormal scores (score 1 or 2) across both groups, affecting 66.5% of the NICU group and 71% of the postpartum ward group. However, while other parameters such as RR and SpO_2_ also showed significantly higher proportions of abnormal scores in the NICU group (*p* < 0.001) for both, these abnormalities were nearly absent in the postpartum ward group. By the second assessment, the proportion of neonates with abnormal BT scores decreased markedly in both groups, although the NICU group maintained a significantly higher rate of abnormalities in BT, RR, and SpO_2_ (all *p* < 0.001). Notably, 50% of neonates in the NICU group continued to have abnormal RR scores at 4–6 hours of life, compared to only 2.6% in the postpartum ward group.

**Table 3:** Distribution of PSU-NEWS scores for each parameter between groups.

Parameter/score	Neonates transferred to NICU n/N (%)	Neonates transferred to postpartum ward n/N (%)	*P*-value
**First PSU-NEWS**			
BT			0.041
Score 0	59/176 (33.5)	586/2015 (29.1)	
Score 1	76/176 (43.2)	1065/2015 (52.9)	
Score 2	41/176 (23.3)	364/2015 (18.1)	
BP			0.122
Score 0	175/176 (99.4)	2015/2015 (100)	
Score 1	-	-	
Score 2	1/176 (0.6)	0/2015 (0)	
HR			0.262
Score 0	173/176 (98.3)	2002/2015 (99.4)	
Score 1	3/176 (1.7)	13/2015 (0.6)	
Score 2	-	-	
RR			<0.001
Score 0	111/176 (63.1)	1882/2015 (93.4)	
Score 1	63/176 (35.8)	133/2015 (6.6)	
Score 2	2/176 (1.1)	0 (0)	
SpO_2_			<0.001
Score 0	166/176 (94.3)	2001/2015 (99.8)	
Score 1	5/176 (2.8)	1/2015 (0.05)	
Score 2	5/176 (2.8)	3/2015 (0.1)	
Neurological			NA
Score 0	176/176 (100)	2015/2015 (100)	
Score 1	-	-	
Score 2	-	-	
**Second PSU-NEWS**			
BT			<0.001
Score 0	145/176 (82.4)	1616/1732 (93.3)	
Score 1	22/176 (12.6)	97/1732 (5.6)	
Score 2	9/176 (5.1)	19/1732 (1.1)	
BP			0.159
Score 0	175/176 (99.4)	1732/1732 (100)	
Score 1	-	-	
Score 2	1/176 (0.6)	0/1732 (0)	
HR			0.015
Score 0	174/176 (98.9)	1731/1732 (99.9)	
Score 1	2/176 (1.1)	1/1732 (0.1)	
Score 2	-	-	
RR			<0.001
Score 0	88/176 (50)	1688/1732 (97.5)	
Score 1	82/176 (46.6)	43/1732 (2.5)	
Score 2	6/176 (3.4)	1/1732 (0.1)	
SpO_2_			<0.001
Score 0	171/176 (97.2)	1732/1732 (100)	
Score 1	4/176 (2.3)	0 (0)	
Score 2	1/176 (0.6)	0 (0)	
Neurological			NA
Score 0	176/176 (100)	1732/1732 (100)	
Score 1	-	-	
Score 2	-	-	

BP, blood pressure; BT, body temperature; HR, heart rate; RR, respiration rate; SpO_2_, oxygen saturation.

The detailed distributions of the first and second PSU-NEWS scores according to the outcome indicators are shown in Table 4.

**Table 4:** Comparison of first and second PSU-NEWS with the outcome indicators.

Variables	Requiring NICU admission		Requiring NIV	
	Yes	No	Yes	No
**First PSU-NEWS**				
n	176	2015	132	2059
Median (IQR) score	1 (1–2)	1 (0–1)^a^	1 (1–2)	1 (0–1)^b^
**Second PSU-NEWS**				
n	176	1732	132	1776
Median (IQR) score	1 (0–1)	0 (0–0)^a^	1 (0–1)	0 (0–0)^b^

IQR, interquartile range; NICU, neonatal intensive care unit; NIV, non-invasive ventilation.

a *p* < 0.001, comparison between neonates who were transferred to the NICU and postpartum wards.

b *p* < 0.001, comparison between neonates who required and did not require NIV.

### 3.1 Score cut-off for predicting NICU admission

**Figure 2:**
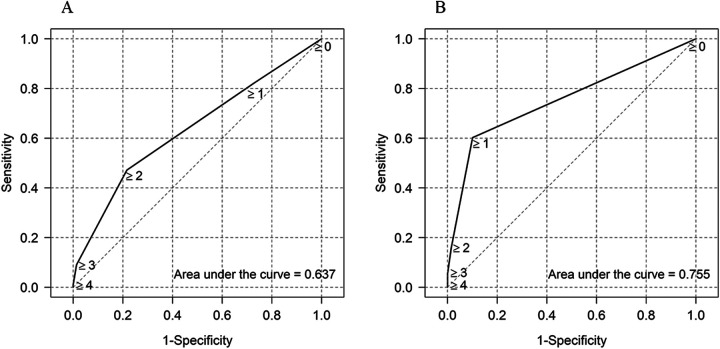
Roc curve of the first **(A)** and second **(B)** PSU-nEWS for predicting nICU admission.

The areas under the ROC curves were 0.637 (95% CI: 0.593–0.681) for the first PSU-NEWS and 0.755 (95% CI: 0.717–0.792) for the second PSU-NEWS. A cut-off score of ≥2 for the first PSU-NEWS yielded a sensitivity of 47%, specificity of 78%, PPV of 16%, NPV of 94%, and an AUC of 0.628 (95% CI: 0.590–0.666). In contrast, a cut-off score of ≥1 for the second PSU-NEWS demonstrated improved performance, with a sensitivity of 60%, specificity of 90%, PPV of 38%, NPV of 96%, and an AUC of 0.751 (95% CI: 0.714–0.788) (Figures 2A,B).

### 3.2 Score cut-off for predicting NIV requirement

**Figure 3:**
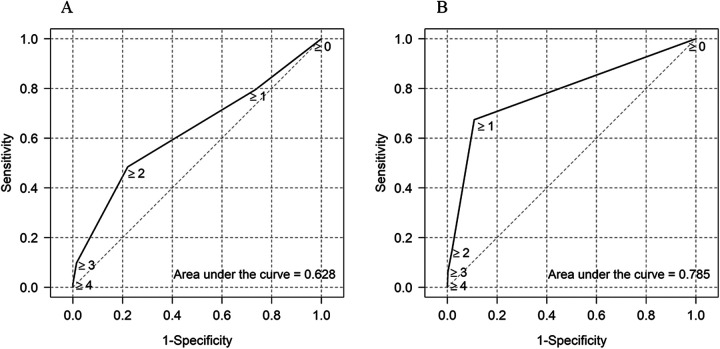
Roc curve of the first **(A)** and second **(B)** PSU-nEWS for predicting NIV requirement.

To predict the requirement for NIV, the AUCs were 0.628 (95% CI: 0.575–0.682) for the first PSU-NEWS and 0.785 (95% CI: 0.744–0.826) for the second PSU-NEWS. Using a cut-off score of ≥2, the first PSU-NEWS showed a sensitivity of 48%, specificity of 78%, PPV of 12%, NPV of 96%, and an AUC of 0.632 (95% CI: 0.589–0.676). In contrast, the second PSU-NEWS, at a cut-off score of ≥1, demonstrated a sensitivity of 67%, specificity of 89%, PPV of 32%, NPV of 97%, and an AUC of 0.783 (95% CI: 0.742–0.824) (Figures 3A,B).

## 4 Discussion

In this study, we evaluated the performance of PSU-NEWS in identifying neonates requiring escalation of care, specifically NICU admission and NIV, in a real-world nursery setting. Our findings demonstrate that the PSU-NEWS has a moderate discriminatory ability for predicting these clinically meaningful outcomes, with improved accuracy in the second assessment performed at 4–6 hours of life.

A key observation of this study is the superior predictive performance of the second PSU-NEWS compared to the first, with the area under the ROC curve increasing from 0.637 to 0.755 for NICU admission and from 0.628 to 0.785 for NIV requirement. While these findings highlight the improved discriminative ability of the second assessment, they also indicate that the first PSU-NEWS, when used in isolation, provides only modest predictive value. However, the role of the initial PSU-NEWS assessment should be interpreted within the context of early neonatal physiology and clinical workflow. The first assessment is performed during a period of rapid physiological transition after birth, when transient abnormalities in respiratory function, oxygenation, and thermoregulation are common and may not necessarily reflect underlying pathology. As such, its primary clinical value is to facilitate early risk stratification and prompt closer observation rather than to provide definitive prediction of adverse outcomes.

By 4–6 hours of life, persistent abnormalities are more likely to represent evolving disease processes rather than transitional physiology, which likely explains the improved discriminative performance observed in the second assessment. These findings support the concept that PSU-NEWS functions most effectively as a dynamic, serial monitoring tool, where repeated assessments provide incremental prognostic value. Importantly, our results do not support eliminating the first assessment from the protocol. Instead, they suggest that reliance on a single early measurement may be insufficient, and that clinical decision-making should incorporate serial evaluations to improve risk stratification and patient safety. The slightly lower gestational age observed in the NICU group, despite similar birth weights, suggests that early-term infants may have increased physiological vulnerability, which could contribute to higher PSU-NEWS scores through clinical manifestations rather than gestational age itself. Furthermore, the high rate of cesarean section (61.5%) in our study, primarily indicated by previous surgical history (45.1%) and cephalopelvic disproportion (15.1%), may further influence these clinical manifestations. Although acute fetal distress, a direct factor for neonatal compromise accounted for only 6.2% of these cases, the physiological challenges associated with surgical delivery, even in non-emergent indications, are known to increase the risk of transient respiratory distress in late-preterm and term neonates, potentially reflected in the initial PSU-NEWS assessments.

The optimal cutoff values identified in this study suggest that lower thresholds at the second assessment provide better sensitivity without substantial loss of specificity. A PSU-NEWS cut-off of ≥1 at the second assessment achieved sensitivities of 60% for NICU admission and 67% for NIV requirement, with high specificities exceeding 89%. In contrast, higher cutoffs at the first assessment demonstrated limited sensitivity, underscoring the risk of under-recognition if early scores were used in isolation. These findings emphasize the importance of interpreting PSU-NEWS results in context and support integrating serial scoring into clinical workflows rather than relying on a single early measurement.

Compared to internationally established neonatal early warning systems, the performance of the PSU-NEWS demonstrates acceptable diagnostic validity while offering distinct practical advantages for busy clinical environments. At the optimal cut-off score ≥2 at 1–2 hours of life to predict NICU admission, the PSU-NEWS achieved a sensitivity of 47% and a specificity of 78%. Importantly, when evaluated at 4–6 hours of life, a cut-off score of ≥1 demonstrated an improved performance, with a sensitivity of 60% and a specificity of 90%. This diagnostic performance is generally lower than that of well-known international tools, such as the Neonatal Trigger System (NTS). Validation studies of the NTS in the United Kingdom and India reported higher sensitivities of 77%–79% and a specificity of 97% at a threshold of ≥2 (6, 7). Similarly, the Whitt-NTS, evaluated multiple times within 48 hours of life in a postnatal ward, demonstrated a sensitivity of 82% and a specificity of 95% (12).

While these international systems yield robust statistical performance, they often require complex clinical inputs, such as blood glucose levels or detailed neurological assessments, which can limit feasibility in resource-constrained or high-workload nursery settings. In contrast, the PSU-NEWS simplifies the required parameters relying strictly on objective vital signs without laboratory or detailed respiratory effort parameters thereby enhancing clinical utility.

Furthermore, our findings contrast with a recent study from Australia, which concluded that a single early warning tool applied across all gestational ages might be ineffective due to its failure to trigger timely escalations compared to standard observation tools (9). The high specificity and improved performance of the PSU-NEWS during sequential assessments suggest that a streamlined, population-specific tool can indeed be effective, particularly when tailored strictly to late preterm and term infants GA ≥35 weeks within a routine nursery environment.

Nevertheless, the lower discriminatory performance and modest AUC of the PSU-NEWS compared to previous systems largely reflect differences in study design and target populations. Unlike many previous studies that focused on selected or high-risk cohorts (12), our study evaluated a large, unselected, and lower-risk nursery population. Additionally, while the initial discriminative ability of the PSU-NEWS was moderate, its performance improved substantially at the second assessment. This improvement parallels findings from other neonatal EWS, reinforcing the critical clinical importance of repeated, serial physiological evaluations over time (12).

To predict the need for NIV, a previous neonatal trigger score study conducted in the United Kingdom demonstrated that reaching predefined score thresholds was associated with a higher likelihood of requiring assisted ventilation with continuous positive airway pressure. Consistent with these findings, our study showed that PSU-NEWS scores at the second assessment were associated with a higher odd of NIV requirement, highlighting the importance of serial assessments for early detection of respiratory deterioration.

Pediatric-oriented systems, such as C-CHEWS, incorporate disease-specific parameters relevant to cardiac patients. The parameters include vital signs (cardiovascular, respiratory, behavioral, and neurological) and staff and family concerns, all requiring intensive monitoring (11). PSU-NEWS prioritizes broadly applicable physiological indicators reflective of common neonatal pathologies encountered in the early postnatal period. Moreover, reliance on routinely monitored parameters enhances the feasibility and sustainability of resource-limited settings. However, one key barrier to the successful implementation of early warning systems is the inadequacy of both human and non-human resources (13). Although the discriminatory performance of PSU-NEWS is modest, particularly at the first assessment, its improved accuracy across serial assessments aligns with findings from other neonatal EWS studies, which emphasize the importance of trend recognition rather than reliance on single time-point measurements (6, 12).

A few limitations should be considered when interpreting our findings. First, the retrospective single-center design may limit generalizability to other institutions with different patient populations or care practices. Furthermore, this study lacked a comparator score, as no formal early warning system was in place at our institution prior to this study. However, the development of the PSU-NEWS represents a significant shift from subjective monitoring to a data-driven, objective approach. Second, the PSU-NEWS was evaluated only in neonates born at least 35 weeks of gestation and weighing at least 1,800 g; therefore, its applicability to premature or low-birthweight infants remains uncertain. Third, while the score demonstrated reasonable specificity, its moderate sensitivity, particularly at the first assessment, suggests that PSU-NEWS should complement, rather than replace, clinical judgment. Fourth, although a general unit policy guided the initiation of NIV in neonates with respiratory distress, the selection of specific NIV modalities was not strictly protocolized and was left to the discretion of the attending neonatologist. This may have introduced inter-physician variability and potential confounding by indication, whereby infants with greater illness severity may have preferentially received more advanced modes of respiratory support. Fifth, the second PSU-NEWS assessment was not available in approximately 13% of neonates, primarily due to early transfer to the postpartum ward or NICU, which may have introduced selection bias, particularly if infants with early clinical deterioration were underrepresented. Finally, residual confounding cannot be excluded, as factors such as clinician decision-making, thresholds for NICU admission, and institutional practices may have influenced both NICU transfer and the initiation of NIV.

Despite these limitations, this study provides important evidence supporting the use of PSU-NEWS as a practical early warning tool in nursery settings, supported by a large sample size. The improved predictive performance observed in the serial assessments underscores the importance of repeated physiological evaluations during the early postnatal period. Future prospective multicenter studies should validate PSU-NEWS across diverse settings, refine the optimal scoring thresholds, and assess its impact on clinical outcomes, including timeliness of intervention, length of stay, and neonatal morbidity.

In conclusion, the PSU-NEWS demonstrated moderate accuracy in identifying neonates requiring NICU admission and respiratory support, with better performance at later postnatal assessments. Its simplicity, integration into routine care, and emphasis on serial evaluation make it a promising tool for early recognition of clinical deterioration and for enhancing patient safety in neonatal care.

## Data Availability

The raw data supporting the conclusions of this article will be made available by the authors, without undue reservation.
